# Targeted Inhibition of miR-221/222 Promotes Cell Sensitivity to Cisplatin in Triple-Negative Breast Cancer MDA-MB-231 Cells

**DOI:** 10.3389/fgene.2019.01278

**Published:** 2020-01-14

**Authors:** Shujun Li, Qun Li, Jinhui Lü, Qian Zhao, Danni Li, Lei Shen, Zhongrui Wang, Junjun Liu, Dongping Xie, William C. Cho, Shaohua Xu, Zuoren Yu

**Affiliations:** ^1^ Research Center for Translational Medicine, Department of Medical Oncology, Shanghai East Hospital, Tongji University School of Medicine, Shanghai, China; ^2^ Department of Maternal and Children Health Management, The Third Hospital of BaoGang Group, Baotou, China; ^3^ Department of Pathological Physiology, Tongji University School of Medicine, Shanghai, China; ^4^ Department of Clinical Oncology, Queen Elizabeth Hospital, Kowloon, Hong Kong; ^5^ Department of Gynecology, Shanghai First Maternity and Infant Hospital, Shanghai, China

**Keywords:** miR-221, miR-222, triple-negative breast cancer, resistance, cisplatin

## Abstract

Cisplatin has been widely used in the treatment of a various types of cancers including triple-negative breast cancer (TNBC) by damaging DNA and inducing apoptosis. However, its anti-cancer effects are often limited due to chemo-resistance, which is one of the main reasons causing cancer relapse and metastasis. To overcome resistance, cisplatin is often used in combination with other drugs or molecules. Our study found that the targeted inhibition of miR-221/222 in MDA-MB-231 cells promoted cisplatin-induced cell apoptosis, and increased the cell sensitivity to cisplatin *in vitro*. Much higher expression levels of miR-221/222 were detected in the cisplatin-resistant MDA-MB-231 cells and in cisplatin-resistant breast cancer patients. The combination chemotherapy of cisplatin with anti-miR-221/222 showed much higher efficiency in suppressing tumor growth in the mice transplanted with MDA-MB-231 cells. In addition, anti-miR-221 and anti-miR-222 showed synergetic effects on improving sensitivity to cisplatin in MDA-MB-231 cells. Suppression of SOCS1-STAT3-Bcl-2 pathway and activation of p53-Pten signaling both contribute to anti-miR-221/222-induced sensitivity to cisplatin in MDA-MB-231 cells. These findings suggest the potential of a novel approach for the combination chemotherapy of cisplatin with small non-coding RNA in treatment of human TNBC.

## Introduction

Breast cancer is the most common cancer and one of the leading causes of cancer-related death in women worldwide. Over 266,120 new cases of invasive breast cancer were diagnosed in women in the United States in 2018 (Siegel et al., 2018). Although traditional treatment and surgery may decrease tumor size, slow down tumor growth and prolong patient survival, the relapse and metastasis, which occurs in 20–30% of women with breast cancer, are still big challenges to influence 10-year survival of patients (Rawindraraj et al., 2018). It has been well reported that the resistance to chemotherapy of cancer cells is one of the main reasons causing relapse and metastasis of cancer (Tryfonidis et al., 2016).

Triple-negative breast cancer (TNBC) is a subtype of breast cancer that based on immunohistochemistry features of estrogen receptor (ER) negative, progesterone receptor (PR) negative and human epidermal growth factor receptor 2 (HER2) negative (Perou, 2011). About 10–20% of breast cancers belong to TNBC subtype, which are more aggressive, higher grade and poorer prognosis than other type of breast cancer (Cleator et al., 2007). Chemotherapy is commonly used for treating TNBC patients in addition to surgical operation, mainly due to lacking of the typical targeted receptors for hormone therapy (Cleator et al., 2007). Platinum-based anti-cancer chemotherapeutic drugs including cisplatin and carboplatin are widely used in treatment of variety of malignancies including TNBC (Dhar et al., 2011; Hu, 2015).

Cisplatin and carboplatin have been used as the first- and second-line chemotherapy for advanced breast cancer (Pentheroudakis et al., 2006], ovarian cancer (Katsumata, 2015) and lung cancer (Griesinger et al., 2019). Cisplatin/carboplatin crosslinks with the purine bases in DNA, causing DNA damage and subsequently inducing apoptosis in cancer cells. However, cancer relapse and metastasis are frequently observed in TNBC patients after cisplatin/carboplatin treatment, mainly due to drug-resistance. To overcome drug resistance and reduce drug toxicity, cisplatin/carboplatin is commonly combined with other drugs such as paclitaxel in treatment of breast cancer (Pentheroudakis et al., 2006; Hu, 2015).

microRNAs (miRNAs) are a class of non-coding small RNAs which are involved in a variety of biological processes including cancer initiation, progression and drug resistance by regulating target gene expression mostly through inhibition of the gene translation and/or cleavage of target gene mRNAs *via* base-pairing with the 3’ untranslated region (3′UTR) (Krek, 2005; Yu, 2010). The miRNA involvement in breast cancer regulation has been well demonstrated by our previous work and others (Iorio, 2005; Yu, 2008; Yu, 2013). We have reported the growth inhibition of MCF-7 cells by miR-17/20 through targeting cyclin D1 (Yu, 2008), which is consistent with the transgenic studies in which miR-17 inhibited cellular growth and proliferation (Shan, 2009). Overexpression of miR-205 and miR-200c inhibited TGF-β-induced EMT in breast cancer (Gregory, 2008). miR-335, miR-206, and miR-126 inhibited breast cancer metastasis and relapse (Tavazoie, 2008). Dicer1, a key regulator for miRNA biogenesis, is induced by cyclin D1 in regulating the miRNA expression profiling and tumorigenesis in human breast cancer (Yu, 2013).

miR-221/222 is a miRNA cluster located on chromosome X regulating human breast cancer (Chen, 2013; Li, 2013). Our previous study has demonstrated the regulation of miR-221/222 to the migration and invasion of TNBC cells (Li, 2014). However, the regulatory mechanisms of miR-221/222 on drug resistance in breast cancer remain unclear. Herein we found the upregulation of miR-221/222 in the cisplatin/carboplatin-resistant breast cancer. Enforced expression of miR-221/222 induced cisplatin resistance in MDA-MB-231 cells. Targeted knockdown of miR-221/222 increased the cellular sensitivity to cisplatin, thereby inducing apoptosis and cell death. SOCS1 is a target gene of miR-221/222 in TNBC. SOCS1-STAT3-Bcl-2 and p53-Pten signaling pathways were found to mediate the miR-221/222-regulated cisplatin sensitivity in MDA-MB-231 cells. The current findings demonstrate a novel function of miR-221/222 in MDA-MB-231 cells, and suggest a novel approach for combination chemotherapy of human TNBC.

## Materials and Methods

### Animals

Animal studies were approved by the Institutional Animal Care and Use Committee of the Tongji University School of Medicine. Six-week-old female nude mice were provided by the Silaike Animal Company (Shanghai, China).

### Cell Lines and Cell Culture

Human breast cancer cell line MDA-MB-231 was purchased from ATCC (Manassas, VA, USA) and maintained in our laboratory. The cisplatin-resistant MDA-MB-231 cell and control were gifts from Dr. Hongfeng Chen at Shanghai University Longhua Hospital. The cisplatin-resistant MDA-MB-231 cells were obtained by discontinuously and gradually increasing dose of cisplatin as described previously (Zhang, 2018). Briefly, MDA-MB-231 wild type cells were stimulated with cisplatin of different concentrations, starting from 100 ng/mL. The survived cells were moved forward to the next stimulation step by increased cisplatin concentration with additional 200–500 ng/mL. After 7-month screening, the survived cells were stably maintained in 4 μg/mL of cisplatin. As validated, the cisplatin-resistant MDA-MB-231 cells had a higher IC_50_ value of cisplatin (19.44 ± 0.89 µg/mL), compared to wild type cells (3.13 ± 0.12 µg/mL) (Zhang, 2018).

DMEM medium containing penicillin and streptomycin (100 mg/L) and 10% fetal bovine serum (FBS) at 37 °C in a humidified environment with 5% CO_2_ was applied for cell culturing.

### Oligos and Transfection

Anti-miR-221, anti-miR-222 and negative control oligos were designed following LNA Oligo Tools and Design Guidelines of Exiqon (Vedbaek, Denmark), and synthesized by GenScript (Nanjing, China). The HiPerFect transfection reagent from Qiagen (Venlo, The Netherlands) was used for cell transfection following the manufacturer’s instructions. Final concentration of 50 nM of the RNA oligo was used for all *in vitro* assays unless stated otherwise.

### miRNA Screening and Real-Time PCR Analysis

Total RNA was extracted with Trizol reagent (Invitrogen, US). First-strand complementary DNA of miRNAs was prepared using the M&G miRNA Reverse Transcription kit (#03002, miRGenes, Shanghai, China) following the manufacturer’s instruction. A Ready-to-Use M&G Human miRNA Profiling qRT-PCR Panel (#04004) covering 365 cancer-related miRNAs was purchased from miRGenes (Shanghai, China). Quantitative real-time PCR based miRNA analysis method was applied to the panels. For miRNA expression analysis, forward primer sequences for these miRNAs were used: miR-221, 5′-agctacattgtctgct-3′; miR-222, 5′-agctacatctggctact-3′; 5s ribosomal RNA, 5′-agtacttggatgggagaccg-3′. All primers were synthesized and purified by GenScript (Nanjing, China). The SYBR GreenMaster Mix was ABI product (#4367660, Applied Biosystem, Life Technologies). The ABI7900 HT Sequence Detection System (Applied Biosystem, Life Technologies) was used for quantitative real time PCR assay. 5S ribosomal RNA was used for normalization.

### Cell Proliferation Assays

For the 3-(4,5-dimethylthiazol-2-yl)-2,5-diphenyltetrazolium (MTT) assay, 2x10^3^ cells/well were seeded into 96-well plate in triplicate. After culturing for 24–72 h as indicated, the cells were stained with MTT solution for 3 h at cell culture condition followed by dissolving with DMSO. The cell growth was determined by measuring OD value at 570 nm.

### Cell Apoptosis Assay

Cells were treated with anti-miR and/or cisplatin, followed by Annexin V staining, and flow cytometry analysis using Annexin V-FITC/IP Assay kit (#401001, Bestbio, China) according to the manufacturer’s instruction.

### Western Blot

Cell lysates (50 μg) were prepared from cells after 48h transfection with anti-miRNA or control, and separated by 10% SDS/PAGE. The proteins were transferred to nitrocellulose membrane. After being blocked in 5% milk (w/v) at room temperature for 1 h, the membranes were incubated at 4°C overnight with primary antibodies (1:1,000). Following 1×PBST washing, the membranes were incubated with secondary antibodies (1:3,000) at room temperature for 1 h followed by ECL staining. The following antibodies were purchased from Santa Cruz Biotechnology for western blot: anti-p27 (sc-776), anti-SOCS1 (sc-9021), anti-c-Myc (sc-40), anti-Pten (sc-7974), anti-p53 (sc-6243), anti-STAT3 (4904s), anti-Bcl-2 (sc-492), and anti-β-actin (sc-47778).

### Combination Effects Analysis

MDA-MB-231 cells were seeded onto 96-well plates for MTT assay. Cells were treated with cisplatin (0, 25, 50, and 100 μM), and a combination of different concentrations of anti-miR-221/222 (0, 25, 50, and 100 nM). After 24 and 48 h incubation, cell viability was assessed with MTT assays. The synergism of cisplatin and anti-miR-221/222 treatment was evaluated by calculating the combination index (CI) value using the cell viability data in 48 h incubation and CalcuSyn software (Biosoft v2.1).

### Preparation of Tumor Burden Mice and Treatment With Cisplatin and Anti-miRNA

1x10^6^ MDA-MB-231 cells were mixed with matrigel, and injected into the fat pad of the fourth mammary gland of nude mice (n = 30). The mice were separated randomly into three groups (n = 10 for each group). The mice in the cisplatin group were treated with cisplatin (5 mg/kg body weight per dose) by intraperitoneal injection weekly. Anti-miRNA negative control (NC) was local injected into tumors with the concentration of 0.25 mg/kg body weight per dose every 72 h for continuous three weeks. The mice in the cisplatin+miR group were treated with cisplatin as same as the cisplatin group, except the tumors were local injected with same amount of anti-miR-221/222 instead. The mice in the control group were intraperitoneally administrated with same volume of 1×PBS solution. The tumors were local injected with anti-miR-NC. The volume of the tumors was measured every other day, and the growth curve of tumors was plotted. All mice were sacrificed at day 23. All tumors were weighted, RNA and proteins were extracted for further analysis.

### miRNA Analysis in the Chemo-Resistant and Chemo-Sensitive Patients With Breast Cancer

The data of breast cancer was from GDC Cancer Datasets (https://portal.gdc.cancer.gov/), and used to analyze the gene expression levels of miR-221 and miR-222 in the chemo-resistant and chemo-sensitive patients. The patients with single drug reaction were included for statistical analysis. A standard way to define how well a cancer patient responds to treatment is based on whether tumors shrink, stay the same, or get bigger according to the response evaluation criteria in solid tumors (RECIST). The patient response can be classified as complete response (the disappearance of all signs of cancer in response to treatment), partial response (a decrease in the size of a tumor, or in the extent of cancer in the body, in response to treatment), progressive disease (the cancer that is growing, spreading, or getting worse), and stable disease (the cancer that is neither decreasing nor increasing in extent or severity). In the current study, we defined complete response and partial response as sensitive, disease progression as resistant, and excluded stable disease.

### Statistical Analysis

Data are presented as mean ± SEM unless stated otherwise. The standard two-tailed Student’s t-test was used for statistical analysis, in which p < 0.05 was considered statistically significant.

## Results

### Upregulation of miR-221/222 in the Cisplatin Resistant TNBC

**Figure 1:**
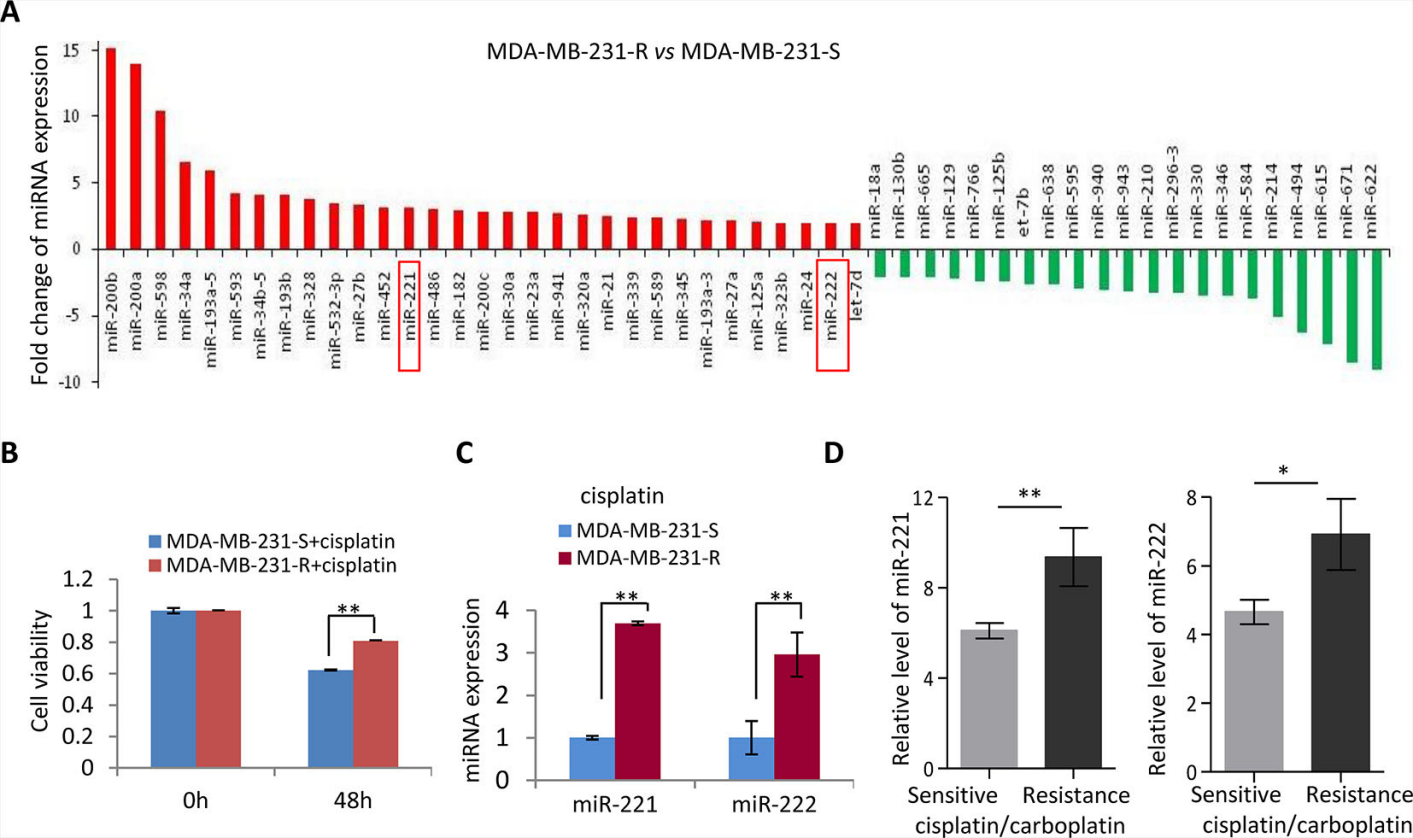
Upregulation of miR-221/222 in the cisplatin-resistant TNBC. **(A)** A miRNA screening identified 31 upregulated miRNAs and 21 downregulated miRNAs with fold change ≥ 2 in MDA-MB-231-R (cisplatin-resistant) cells compared to MDA-MB-231-S (cisplatin-sensitive) cells. **(B)** Validation of the sensitivity to cisplatin of MDA-MB-231-R and MDA-MB-231-S cells by 48h-treatment with 20 μM cisplatin. **(C)** Validation of the upregulation of miR-221 and miR-222 in MDA-MB-231-R cells. **(D)** TCGA database showed higher level of miR-221 and miR-222 in the cisplatin/carboplatin-resistant breast cancer patients (n = 3) compare to -sensitive patients (n = 7). Values are equal to mean ± SEM. *p < 0.05, **p < 0.01.

In view of the relapse and metastasis of breast cancer partly due to resistance to chemotherapy, a miRNA expression screening was performed in the cisplatin-resistant and sensitive MDA-MB-231 (MDA-MB-231-R and MDA-MB-231-S) cells (**Figure 1A**) to determine the key genes regulating cisplatin resistance in MDA-MB-231 cells. Cisplatin resistance of MDA-MB-231-R was confirmed by 48h-treatment with cisplatin (**Figure 1B**). A subset of miRNAs was identified showing differential expression in the cisplatin-resistant MDA-MB-231 cells, including upregulation of miR-200a/b, miR-34a/b, miR-221/222, and downregulation of miR-622 and miR-671 (**Figure 1A**), in which some miRs including the miR-200 family and the miR-34 family have been well demonstrated for the cancer-regulating function. In combination with the miRNA expression pattern in the patients resistant to platinum, we focused on oncogenic miR-221/222 cluster for further analysis. The upregulation of miR-221/222 in the MDA-MB-231-R cells were validated (**Figure 1C**). According to an analysis on GDC cancer database, both miR-221 and miR-222 showed significant upregulation in the cisplatin/carboplatin-resistant breast cancer patients compared to cisplatin/carboplatin-sensitive controls (**Figure 1D**).

### Inhibition of miR-221/222 Promoted Cell Sensitivity to Cisplatin in MDA-MB-231 Cells

**Figure 2:**
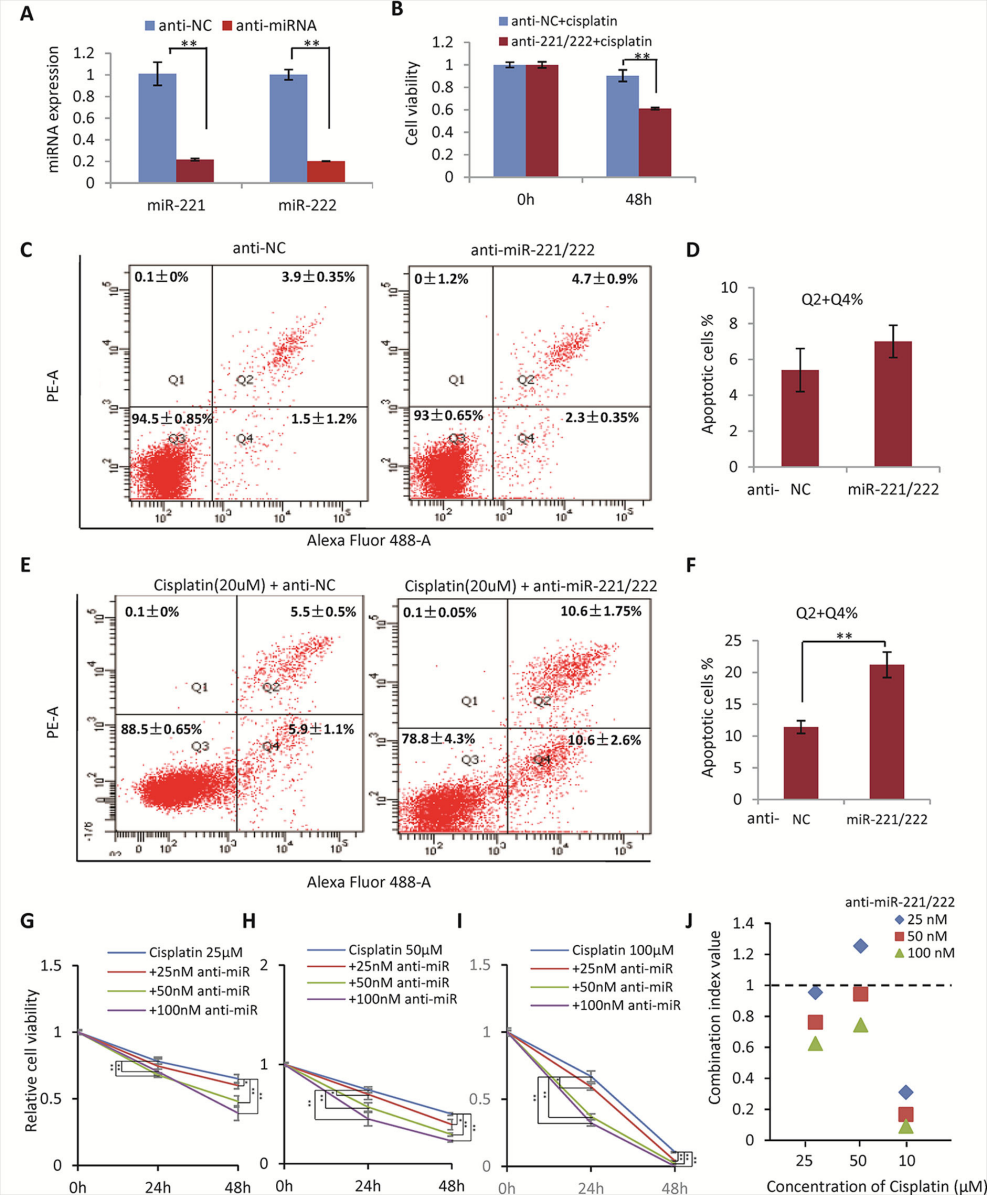
Inhibition of miR-221/222 Promoted Cell Sensitivity to Cisplatin in MDA-MB-231 cells. **(A)** Knockown of miR-221 and miR-222 by transfection with 50 nM anti-miR-221 and anti-miR-221 in MDA-MB-231-R cells, respectively. **(B)** Knockdown of miR-221/222 significantly promoted cellular sensitivity to cisplatin (20 μM, 48 h) from 15% (NC group) to 40% (anti-miR group) in MDA-MB-231-R cells. **(C)** Knockdown of miR-221/222 did not induce apoptosis in MDA-MB-231 cells without cisplatin stimulation, analyzed with annexin V staining. **(D)** Quantitative analysis of apoptotic cells in C. **(E)** Knockdown of miR-221/222 significantly increased the apoptotic cell proportion from ~12% (NC group) to ~22% (anti-miR group) after treatment with 20 μM cisplatin. **(F)** Quantitative analysis of apoptotic cells in E. **(G–I)** MTT assays showed the cell viability after treatment with different concentrations of cisplatin (25 **(G)**, 50 **(H)**, and 100 **(I)** μM) combined with 25, 50, and 100 nM of anti-miR-221/222 in MDA-MB-231 cells, respectively. **(J)** The CI values indicated synergism of cisplatin and anti-miR-221/222 in treatment of MDA-MB-231 cells. Data are mean ± SEM (n = 3). *p < 0.05, **p < 0.01.

In view of the upregulation of miR-221/222 in the cisplatin/carboplatin-resistant breast cancer, the function of miR-221/222 was determined by targeted knockdown in the cisplatin-resistant MDA-MB-231 cells (**Figure 2A**), followed by cisplatin treatment and cell viability examination. As shown in **Figure 2B**, knockdown of miR-221/222 using anti-miR-221 and anti-miR-222 inhibitors significantly promoted cellular sensitivity to cisplatin (20 μM, 48 h) from 15% (NC group) to 40% (anti-miR group) in the MDA-MB-231-R cells.

In addition, annexin V staining and flow cytometry analysis were applied to detect apoptosis of MDA-MB-231 cells with or without cisplatin treatment before and after anti-miR-221/222 transfection. Anti-miR-221/222 did not induce apoptosis in the cells without cisplatin stimulation (**Figures 2C, D**). However, anti-miR-221/222 significantly increased the apoptotic cell proportion from ~12% (NC group) to ~22% (anti-miR group) after treatment with 20 μM cisplatin (**Figures 2E, F**), indicating anti-miR-221/222 is able to promote cell sensitivity to cisplatin in MDA-MB-231.

In order to further determine the combination effects between cisplatin and anti-miR-221/222 in suppressing breast cancer cell viability, MTT assays were applied to MDA-MB-231 cells treated with different concentrations combination of cisplatin and anti-miR-221/222. The synergism of cisplatin and anti-miR-221/222 treatment was evaluated by calculating the combination index (CI) in which the CI value <1 was defined as synergism. As shown in **Figures 2G–I**, 25, 50, and 100 μM of cisplatin was combined with 25, 50, and 100 nM of anti-miR-221/222 to treat MDA-MB-231 cells, respectively. The cell viability was inhibited by cisplatin alone and combination with anti-miR-221/222, which showed dose-dependent inhibition. The CI values indicated a good synergism of cisplatin and anti-miR-221/222 in treatment of MDA-MB-231 cells (**Figure 2J**).

### Anti-miR-221 and Anti-miR-222 Have Synergetic Effects on Promoting Sensitivity to Cisplatin in MDA-MB-231 Cells

**Figure 3:**
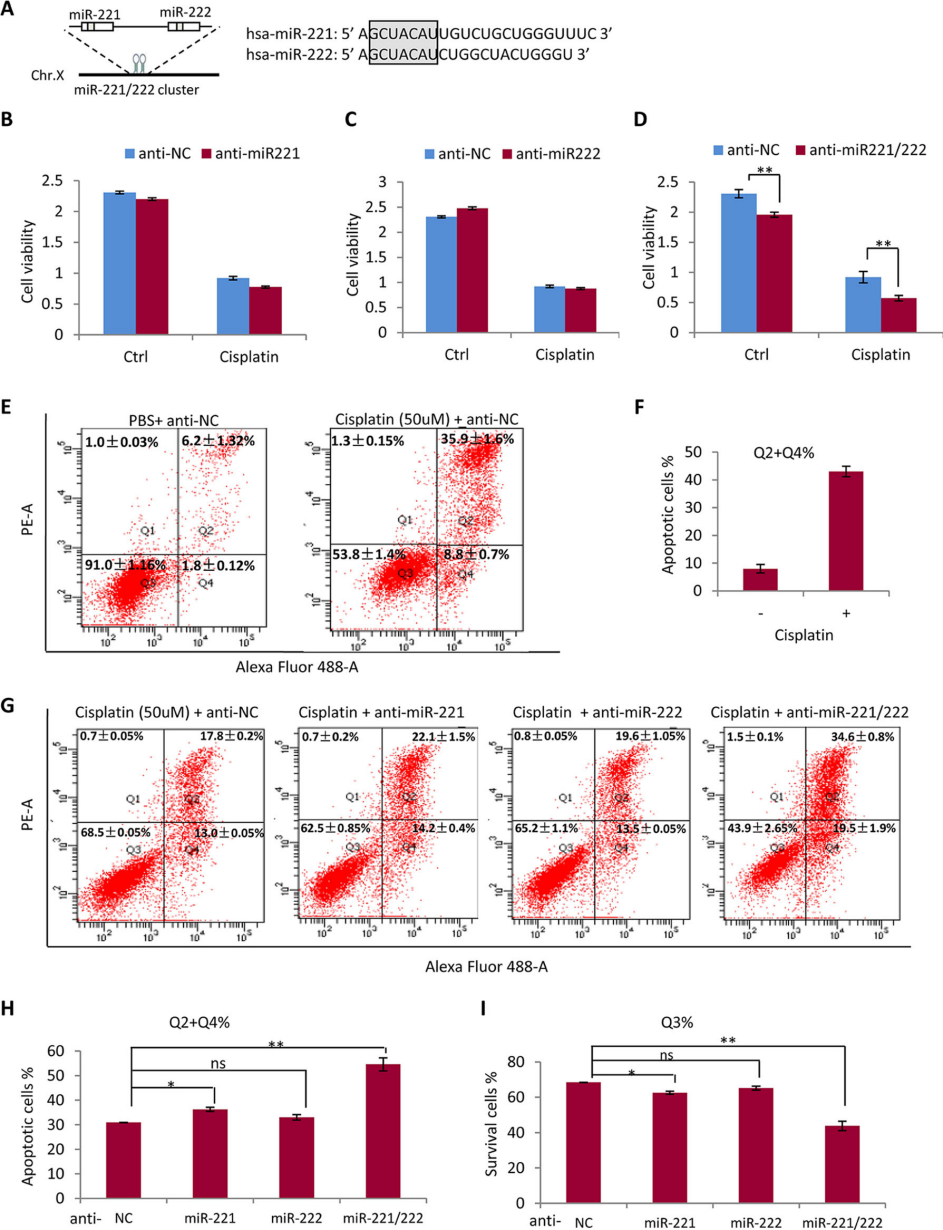
Anti-miR-221 and anti-miR-222 have synergetic effects on promoting sensitivity to cisplatin in MDA-MB-231 cells. **(A)** miR-221 and miR-222 share same seed sequence. **(B**, **C)** Anti-miR-221 **(B)** or anti-miR-222 **(C)** only did not show significant effect on sensitivity to cisplatin in MDA-MB-231 cells. **(D)** Combination of anti-miR-221 with anti-miR-222 significantly promoted the efficiency of cisplatin in suppressing the growth of MDA-MB-231 cell. **(E)** 50 μM of cisplatin inhibited cell growth and induced cell apoptosis in MDA-MB-231 cell. **(F)** Quantitative analysis of apoptotic cells in E. **(G)** Flow cytometry analysis after annexin V staining in MDA-MB-231 cells further demonstrated the synergetic effects of anti-miR-221 and anti-miR-222 to increase cell sensitivity to cisplatin. **(H)** Quantitative analysis of apoptotic cells in G. **(I)** Quantitative analysis of survived living cells in G. Data are mean ± SEM (n = 3). *p < 0.05, **p < 0.01, ns, non significant.

Since miR-221 and miR-222 share same seed sequence and may share same target genes (**Figure 3A**), it is necessary to determine the compensation or synergism of miR-221 and miR-222 in regulating drug sensitivity in TNBC. As shown in **Figures 3B, C**, application of anti-miR-221 or anti-miR-222 only did not show significant effect on cellular sensitivity to cisplatin, while combination of anti-miR-221 with anti-miR-222 significantly promoted the efficiency of cisplatin in suppressing the growth of MDA-MB-231 cells (**Figure 3D**), indicating the requirement of knockdown both miR-221 and miR-222 to sensitize the cisplatin activity in treatment of MDA-MB-231 cells. miR-221 and miR-222 showed functional compensation each other mainly due to sharing same seed sequence and same target genes. As such, anti-miR-221 and anti-miR-222 showed synergetic effects on promoting sensitivity to cisplatin (**Figure 3D**).

Consistently, flow cytometry analysis after annexin V staining in MDA-MB-231 cells further demonstrated the synergetic effects of anti-miR-221 and anti-miR-222. As shown in **Figures 3E**, **F**, treatment with high concentration (50 μM) of cisplatin for 48 h obviously inhibited cell growth and induced cell apoptosis. Combined application of anti-miR-221 and anti-miR-222 to MDA-MB-231 cells significantly increased the sensitivity to cisplatin to 55% from 30% in NC group (**Figures 3G–I**).

### Combination Chemotherapy of Cisplatin With Anti-miR-221/222 in Suppression of Tumor Growth *In Vivo*

**Figure 4:**
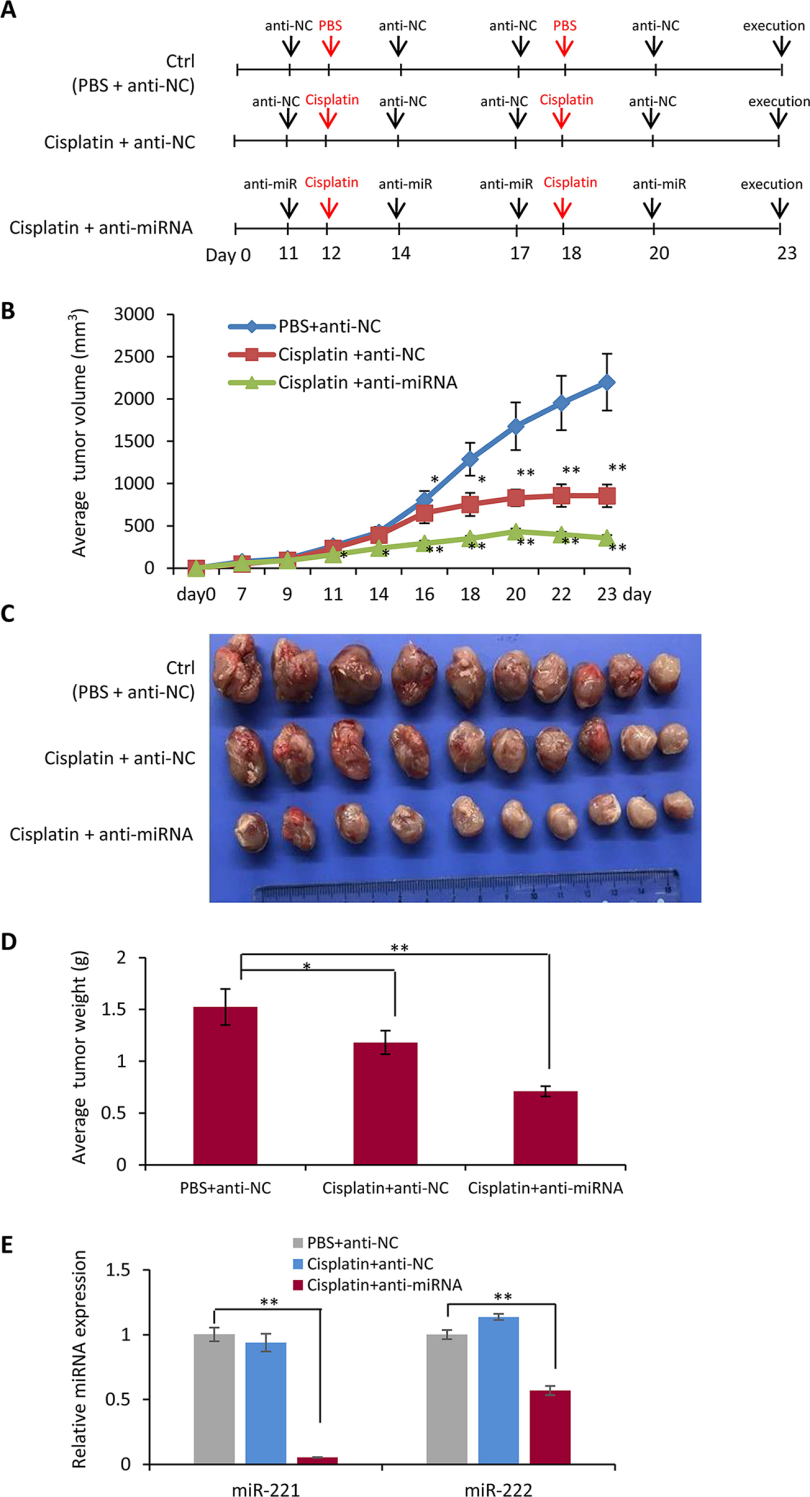
Combination chemotherapy of cisplatin with anti-miR-221/222 in suppression of tumor growth *in vivo*. **(A)** Schematic representation of the procedure for combination of cisplatin with anti-miR-221/222 in treatment of TNBC tumor burden mice developed by transplantation of MDA-MB-231 cells to the fat pad of the fourth mammary gland of nude mice. **(B)** Tumor growth curves after treatment with cisplatin or cisplatin +anti-miR-221/222, compared to control group treated with PBS and anti-miR NC. **(C)** Images of tumors from the three groups of mice. **(D)** Average weight of the tumors in the three groups of mice. **(E)** Expression levels of miR-221 and miR-222 in tumors from the three groups of mice. Values are equal to mean ± SEM (n = 10). *p < 0.05, **p < 0.01.

In order to further determine the function of miR-221/222 in regulating TNBC chemotherapy, tumor burden mice were developed by transplantation of MDA-MB-231 cells to the fat pad of the fourth mammary gland of nude mice, followed by cisplatin treatment through intraperitoneal injection and anti-miR-221/222 application through local delivery into the tumors as shown in **Figure 4A**. Tumor growth curves showed inhibition of tumor growing by cisplatin from day 16 on after the cell transplantation (cisplatin group *vs* control group, **Figure 4B**). Application of anti-miR-221/222 significantly promoted the anti-tumor effects of cisplatin from day 11 on (cisplatin+miR group *vs* control group, **Figure 4B**). In three weeks after cell transplantation, the mice were sacrificed, and the tumors were taken out and weighted. As shown in **Figures 4C, D**, cisplatin+miR group had the smallest tumor size and the lightest tumor weight compared to cisplatin and control groups. The gene expression analysis demonstrated the knockdown of miR-221 and miR-222 in the tumors from cisplatin+miR group (**Figure 4E**), indicating the effective delivery of anti-miR-221/222 into tumor cells by local injection.

### SOCS1-ATAT3-Bcl2 and p53-Pten Involved in the miR-221/222 Regulation of Cisplatin Sensitivity

**Figure 5:**
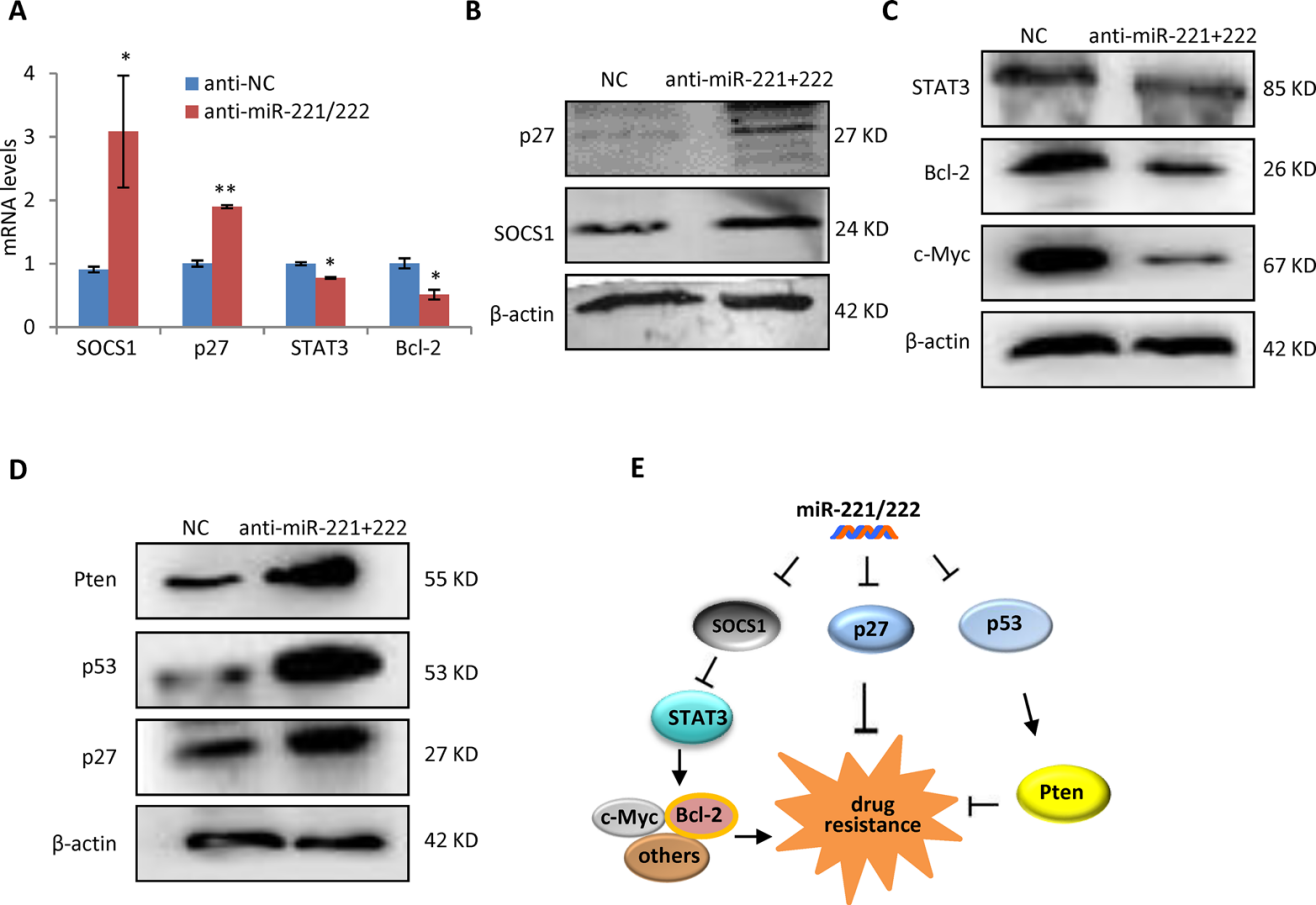
SOCS1-STAT3-Bcl-2 pathway and p53-Pten signaling involved in the miR-221/222 regulation of cisplatin sensitivity in TNBC. **(A)** QRT-PCR analysis of mRNA levels of SOCS1 and p27, two target gene of miR-221/222 in TNBC, and their downstream genes including STAT3 and Bcl-2 in MDA-MB-231 cells after treatment with anti-miR-221/222 or control. **(B)** Western blot showing upregulation of p27 and SOCS1 after knockdown of miR-221/222 in MDA-MB-231 cells. β-actin served as loading control. **(C)** Western blot showing suppression of STAT3, Bcl-2 and c-Myc after knockdown of miR-221/222 in MDA-MB-231 cells. β-actin served as loading control. **(D)** Western blot showing upregulation of p53 and Pten after knockdown of miR-221/222 in MDA-MB-231 cells. β-actin served as loading control. **(E)** Schematic diagram of the mechanism through which miR-221/222 regulates cell sensitivity to cisplatin in MDA-MB-231 cells. Data are mean ± SEM (n = 3). *p < 0.05, **p < 0.01.

In order to determine the mechanism through which miR-221/222 regulates cisplatin sensitivity in TNBC, p27 and SOCS1, two targets genes of miR-221/222 identified by our previous work (Li, 2014), were analyzed in expression at the both mRNA and protein levels in MDA-MB-231 cells before and after transfection with anti-miR-221/222. Upregulations of p27 and SOCS1 were associated with miR-221/222 knockdown (**Figures 5A, B**). STAT3, as a widely-confirmed downstream target gene of SOCS1 was further analyzed, showing inhibition in expression by anti-miR-221/222 (**Figures 5A–C**). STAT3 transcriptionally regulates expression of a set of downstream genes including c-Myc and Bcl2 in control of tumorigenesis and chemo-resistance in cancer (Fathi et al., 2018; Gao et al., 2019). Our further analysis indicated the decrease of c-Myc and Bcl2 in expression by anti-miR-221/222, which is consistent with the upregulation of SOCS1 and downregulation of STAT3 (**Figures 5A–C**). Additional analysis on the key regulators of apoptosis revealed upregulation of p53 and Pten in MDA-MB-231 cells after knockdown of miR-221/222 (**Figure 5D**), which may partly contribute to the cisplatin plus anti-miR-221/222 induced cell apoptosis and cell death. These results suggest that suppression of SOCS1-STAT3-Bcl-2 pathway and/or activation of p53-Pten signaling involve in regulation of anti-miR-221/222-induced sensitivity to cisplatin in MDA-MB-231 cells (**Figure 5E**).

## Discussion

Cisplatin is one of the first- and second-line drugs widely used to treat various types of solid tumors. It kills cancer cells by damaging DNA, inhibiting DNA synthesis and subsequently inducing apoptosis. There are two major pathways of apoptotic cell death (Nunez et al., 1998; D’Arcy, 2019). The extrinsic pathway is activated by the tumor necrosis factor-α (TNFα) family and death-inducing signaling complex (DISC). The intrinsic pathway is initiated by cellular stresses, such as DNA damage to activate the cytochrome C-caspase-9-APAF-1 signaling and form an active apoptosome complex (Nunez et al., 1998; Dasari and Tchounwou, 2014; D’Arcy, 2019). Bcl-2 family proteins regulate DNA damage-induced apoptosis (Takahashi and Loo, 2004). Cisplatin-induced genotoxic stress in cancer cells activates multiple signal transduction pathways including the intrinsic apoptotic pathway, resulting in cellular apoptosis (Basma, 2005; Dasari and Tchounwou, 2014). In this study, we demonstrated a mechanism through which miR-221/222 promotes SOCS1-STAT3-Bcl-2 signaling pathway in regulating the cell sensitivity to cisplatin in human TNBC cells(**Figure 5E**).

In addition to inducing DNA damage, chemotherapy often activates the DNA repair system, which has been considered as a main reason causing anticancer drug resistance (Gottesman et al., 2002). For example, cisplatin damages DNA crosslinks and induces nucleotide excision repair and homologous recombination, which in turn leads to cell resistance to cisplatin (Dasari and Tchounwou, 2014; Bonanno et al., 2014). As Such, cancer cells that lack DNA repair abilities are more sensitive to platinum-based chemotherapy. TNBC and high-grade serous ovarian cancers are characterized by a wide range of genomic alterations which are believed partly due to defects in DNA repair such as homologous recombination deficiency (HRD) (Silver et al., 2010; Watkins et al., 2015). HRD is mainly caused by mutations in genes, such as BRCA1, BRCA2, RAD51 and PALB2, which are required for DNA double strand break repair by homologous recombination (Pellegrini et al., 2002; Timms et al., 2014; Watkins et al., 2015). Emerging evidence has demonstrated the correlation between HRD and genomic instability with sensitivity to platinum-based chemotherapy in TNBC (Telli et al., 2016). Our results identified a non-coding RNA family in regulating DNA damage response and response to platinum-based chemotherapy in TNBC, which adds a node to the regulatory network of HDR and platinum sensitivity in TNBC.

In order to overcome resistance, cisplatin is often used in combination with other drugs. For example, cisplatin combination chemotherapy with paclitaxel and fluorouracil has been widely applied to patients with breast cancer (Cancello, 2015; Elserafi et al., 2018). Furthermore, combination chemotherapy of cisplatin with other molecules is also considered as the approaches to overcome drug-resistance. Inhibition of DNA repair pathway could sensitize cancer cells to drug, and therefore increase chemotherapy efficiency. In the current study, anti-miR-221/222 was confirmed in a TNBC cell line to reverse DNA damage repair and promote cell sensitivity to cisplatin. The combination chemotherapy of cisplatin with anti-miR-221/222 showed higher efficiency to suppress tumor growth in the mice model carrying TNBC tumors.

SOCS1 is a member of the suppressor of cytokine signaling family which negatively regulates cytokine signal transduction through inhibiting the Jak/STAT pathway (Slattery et al., 2013). Downregulation of SOCS1 has been confirmed in various cancer types including TNBC (Slattery et al., 2013; Li, 2014). The STAT3 signaling pathway regulates cancer cell proliferation and apoptosis (Xie, 2004). A recent study found that the inactivation of STAT3 is able to rescue chemo-resistance in breast cancer (Kuo, 2017; Cheng, 2018). We have previously demonstrated SOCS1 as a target gene of miR-221/222 in regulating cell migration and invasion in TNBC cells (Li, 2014). This study demonstrated a novel function of miR-221/222-SOCS1 in regulating cisplatin resistance in TNBC cells *via* activating STAT3 signaling pathway.

In addition to SOCS1-STAT3-Bcl-2 signaling pathway, p53 and Pten also showed regulation by miR-221/222 in MDA-MB-231 cells (**Figure 5C**). p53 transactivates genes involved in the cell cycle, DNA repair and apoptosis. p53 regulates cisplatin-induced DNA damage response and cell death by several mechanisms including the induction of Pten and/or inhibition of AMPK (Basu and Krishnamurthy, 2010). Pten has been frequently reported to involve in the regulation of chemo-resistance, including cisplatin resistance in human TNBC (Wu et al., 2019). As such, the current results suggest that the suppression of SOCS1-STAT3-Bcl-2 pathway and activation of p53-Pten signaling both contribute to anti-miR-221/222-induced cell sensitivity to cisplatin in TNBC.

On a whole, the current study demonstrated a novel function of miR-221/222 in regulating chemo-resistance of TNBC cells. It suggests a novel approach for combination chemotherapy of cisplatin with small non-coding RNA in the treatment of human TNBC.

## Data Availability Statement

Publicly available datasets were analyzed in this study. This data can be found here: https://portal.gdc.cancer.gov/repository?filters = %7B%22op%22%3A%22and%22%2C%22content%22%3A%5B%7B%22op%22%3A%22in%22%2C%22content%22%3A%7B%22field%22%3A%22cases.case_id%22%2C%22value%22%3A%5B%22dcd5860c-7e3a-44f3-a732-fe92fe3fe300%22%5D%7D%7D%2C%7B%22op%22%3A%22in%22%2C%22content%22%3A%7B%22field%22%3A%22files.data_category%22%2C%22value%22%3A%5B%22Clinical%22%5D%7D%7D%5D%7D&searchTableTab = files.

## Ethics Statement

This study was carried out in accordance with the recommendations of the Experimental Animal Use Regulation from the Institutional Animal Care and Use Committee of the Tongji University School of Medicine. The protocol was approved by the Institutional Animal Care and Use Committee of the Tongji University School of Medicine.

## Author Contributions

SL, QL, DL, and JHL performed cellular and molecular experiments. QZ and DX performed bioinformatics analysis. JJL, LS, and ZW did animal experiments. WC, SX, and ZY designed the experiments and wrote the paper.

## Funding

This work was supported by grant from the National Key Research and Development Program of China Stem Cell and Translational Research (2016YFA0101202); grants 81502288, 81572593, 81772762 and 81772810 from the National Natural Science Foundation of China.

## Conflict of Interest

The authors declare that the research was conducted in the absence of any commercial or financial relationships that could be construed as a potential conflict of interest.
